# Molecular cytogenetics provides insights into heterochromatin composition and karyotypic evolution in species of the *Plebeia* group (Apidae: Meliponini)

**DOI:** 10.1186/s13039-026-00775-2

**Published:** 2026-06-16

**Authors:** Cristiano Lula Campos, Gisele Amaro Teixeira, Denilce Meneses Lopes, Ana Maria Waldschmidt

**Affiliations:** 1 Departamento de Ciências Biológicas, Universidade Estadual do Sudoeste da Bahia, Campus de Jequié, Jequié, 45200000 Bahia Brazil; 2 Laboratório de Citogenética de Insetos, Departamento de Biologia Geral, Universidade Federal de Viçosa, Campus Universitário, Viçosa, 36570900 Minas Gerais Brazil

**Keywords:** C_0_*t*-DNA, FISH, Heterochromatin, Microsatellite, Stingless bee

## Abstract

**Background:**

The *Plebeia* group, including *Plebeia*, *Lestrimelitta*, and *Friesella*, requires clearer taxonomic resolution. *Plebeia* is the polyphyletic: species in clade II are more closely related to *Friesella* and *Lestrimelitta* than to their congeners in clade I, which are more restricted to the Amazon biome, and Central and North America. Molecular cytogenetic studies in Meliponini have shown the important role of heterochromatin in karyotypic evolution and species diversification. In this study, we compared heterochromatic repetitive sequences in the *Plebeia* group to better understand its evolutionary patterns and diversification.

**Results:**

For this, we hybridized a probe of highly and moderately repetitive DNA from the *Friesella schrottkyi* genome (here called FSch-C_0_*t* probe) onto the chromosomes of *F. schrottkyi*, *Lestrimelitta limao*, and some *Plebeia* species from clade II to investigate whether repetitive sequences constituting heterochromatin are shared among species. Additionally, we mapped the microsatellite (GA)_*n*_ in four *Plebeia* species from clade II as a control experiment, as it typically shows general euchromatic hybridization patterns in stingless bees. The FSch-C_0_*t* probe labeled all heterochromatin in *F. schrottkyi* and was restricted to the centromeric heterochromatin in analyzed *Plebeia* species from clade II, indicating the sharing of repetitive sequences in part of the heterochromatin between these two genera. However, the FSch-C_0_*t* did not yield signals in the AT-rich regions of *L. limao*, which likely correspond to heterochromatin, suggesting a variation in the heterochromatic composition compared to that of *F. schrottkyi* and analyzed *Plebeia* species from clade II. In addition, FSch-C_0_*t* signals were detected in some terminal regions of the chromosomes in *Plebeia* spp. and *L. limao*, potentially corresponding to telomeric sequences. Microsatellite (GA)_*n*_ was located on one of the chromosomal arms in the euchromatic regions of the four *Plebeia* taxa, following a trend reported for other Meliponini.

**Conclusion:**

Our data reveal a dynamic evolution of heterochromatic composition in the *Plebeia* group, with hypotheses regarding its diversification discussed in light of available phylogenies.

**Supplementary Information:**

The online version contains supplementary material available at https://doi.org/10.1186/s13039-026-00775-2.

## Introduction

Repetitive sequences play a crucial role in the diversification and plasticity of eukaryotic genomes, potentially contributing to environmental adaptations and speciation [1, 2, 3]. In stingless bees (tribe Meliponini), molecular cytogenetic studies using fluorescence *in situ* hybridization (FISH) with repetitive DNA probes have facilitated comparisons of heterochromatic composition between related taxa, revealing its pivotal role in karyotypic evolution and species diversification [4, 5, 6, 7, 8, 9, 10, 11]. For instance, Lopes et al. [4] showed that repetitive sequences are shared among the chromosomes within each species—*Melipona rufiventris* and *Tetragonisca fiebrigi* —although the heterochromatic composition differed between the two genera. Similarly, a repetitive sequence is distributed throughout the heterochromatin in *Tetragonisca angustula* and *T. fiebrigi*, which was not shared with other Meliponini genera, such as *Partamona*, *Nannotrigona*, *Frieseomelitta*, *Melipona* and *Austroplebeia* [7].

In addition, within the *Melipona* genus, heterochromatic repetitive sequences are shared among species within each subgenera with high heterochromatic content (*Michmelia* and *Melikerria*), but not between different subgenera. These differences suggest independent sequence amplification in each subgenus, reflecting the diversification of the *Melipona* [6, 8]. Cytogenomic analyses revealed that the heterochromatin of *Melipona scutellaris* (subgenus *Michmelia*), is primarily composed of exclusive satellite DNA (here referred to as satDNA), which is highly abundant (approximately 38%) in the genome of this species. Conversely, *Melipona quadrifasciata*, from the subgenus *Melipona sensu stricto*, which is characterized by low heterochromatic content, has its most abundant exclusive satDNA (approximately 0.9%) confined to the pericentromeric heterochromatin of a single chromosome pair [9].

Similarly, in the *Trigona* genus, a specific satDNA is shared among closely related species of the same phylogenetic clade, but absent in the sister clade, indicating independent evolution of heterochromatic composition within this genus [10]. More recently, Vignati et al. [11] revealed for the first time in Meliponini, that a satDNA family (FvarSat01-306) is shared across different genera within the largest clade of Neotropical Meliponini, being present in the part or in the whole heterochromatic region.

Building on these findings, the *Plebeia* group, which includes the genera *Plebeia*, *Friesella*, and *Lestrimelitta*, provides a suitable framework to investigate how the study of repetitive sequences within heterochromatin can contribute to understanding species genome diversification and karyotypic evolution, given its complex taxonomic status and karyotypic variability. Molecular phylogenies have shown that the monophyletic genera *Friesella* and *Lestrimelitta* are positioned between two independent clades of *Plebeia* (I and II), indicating that the latter is polyphyletic. Clade I comprises *Plebeia* species that are more restricted to the Amazon biome, as well as Central and North America, and is sister to the clade containing *Friesella*, *Lestrimelitta*, and other *Plebeia* species (clade II) [12, 13, 14]. Cytogenetic data available for the *Plebeia* group reveal karyotypic variability. *Lestrimelitta* has 2n = 28 chromosomes, whereas *Friesella* and *Plebeia* have 2n = 34 [15]. Moreover, some *Plebeia* species from clade II exhibit up to two B chromosomes in their karyotypes [16, 17]. Other cytogenetic features also vary both among and within genera, including the number and chromosomal location of ribosomal gene sites, as well as the distribution of microsatellites, which may occur on a single euchromatic arm or on both chromosome arms [18, 19]. Despite this diversity, the contribution of heterochromatin and repetitive sequences to karyotype differentiation and taxonomic divergence within the *Plebeia* group remains poorly understood. Addressing this gap may help clarify the evolutionary processes underlying the complex relationships among these genera.

Furthermore, FISH experiments have also revealed that other types of repetitive sequences are predominantly located in the euchromatin of stingless bees, such as di-, tri-, and pentanucleotide microsatellites, assisting in discussions about the importance of chromosome fusions in the group [19]. The C_0_*t*-DNA renaturation kinetics technique has enabled the comparison of heterochromatic composition among species, allowing us to determine whether repetitive sequences are shared in this chromosomal region and gain a better understanding of their evolutionary patterns [20, 21, 22, 23, 24]. Therefore, in this study, we produced a probe containing a pool of moderately and highly repetitive sequences from the genome of *Friesella schrottkyi* using the C_0_*t*-DNA renaturation kinetics technique, herein referred to as the Fsch-C_0_*t* probe. This probe was hybridized on the chromosomes of the same species, of the *Lestrimelitta limao*, and some *Plebeia* species from clade II to verify whether these repetitive sequences were shared in the heterochromatin of these genera. This approach allowed us to test the hypothesis if repetitive sequences are conserved across genera within the *Plebeia* group. We related our results to the available phylogenies and discussed evolutionary scenarios regarding heterochromatic composition and karyotypic evolution in this group. We also performed physical mapping of the (GA)_n_ microsatellite in four *Plebeia* taxa to determine whether its chromosomal location follows the general euchromatic hybridization pattern observed in other stingless bees.

## Methods

Samples of stingless bee species *F. schrottkyi*, *L. limao*, and *Plebeia* clade II representatives *Plebeia lucii*, *Plebeia* cf. *mosquito*, *Plebeia* aff. *droryana* 1, *Plebeia* aff. *droryana* 2, and *Plebeia* aff. *flavocincta* were used in this study for molecular cytogenetic analysis (see Table S1). These *Plebeia* species from clade II were selected due to the availability of cytogenetic material obtained from previous studies, which also described their karyotypes (see Table S1). Specimens of the *Plebeia* were identified by Dr. Hugo Werneck. Our evolutionary interpretations are based on the phylogenetic framework proposed by Werneck [13], as well as on recent findings by Lepeco et al. [14]. Unfortunately, no specimens from *Plebeia* clade I were available for this study. Therefore, our sampling focused on clade II species, allowing comparative analyses but not covering the full diversity of the *Plebeia* group. The mitotic metaphases from the stingless bee species were obtained from cerebral ganglia of the larvae after meconium elimination, according to the protocol described by Imai et al. [25].

The Fsch-C_0_*t* probe enriched with moderately and highly repetitive sequences from *F. schrottkyi* was obtained according to the protocol described by Zwick et al. [26], with modifications in the DNA precipitation step by Cunha et al. [6]. The total DNA from *F. schrottkyi* was isolated according to Waldschmidt et al. [27]. Briefly, genomic DNA was diluted to a final concentration of 100–500 ng/µL in 0.3 mol/L sodium chloride (NaCl). The sample was autoclaved for 15 min at 121 °C (1.4 atm) to generate fragments between 100 and 1000 bp. The DNA was then denatured at 95 °C for 10 min and re-annealed at 65 °C for 10 min. Subsequently, the DNA was digested with nuclease S1 (Promega) at 37 °C for 8 min. The DNA was precipitated by adding 0.1 volume of 3 M sodium acetate and 1 volume of 2-propanol. A sample was checked on a 0.8% agarose gel, confirming fragment sizes ranging from 50 to 500 bp. The Fsch-C_0_*t* probe was indirectly labeled with digoxigenin-11-dUTP using the kit DIG-Nick Translation Mix (Roche Applied Science, Mannheim, Germany). FISH signals were detected with anti-digoxigenin-rhodamine (Roche Applied Science), following the manufacturer’s instructions.

In addition, the microsatellite (GA)_15_ was used as a probe directly labeled with Cyanine-3 (Cy3) at the 5’ terminus (Sigma, St. Louis, MO, USA). Due to the limited availability of cytogenetic slides for the analyzed species, we chose to use only the (GA)₁₅ microsatellite motif, which has already been extensively tested and shown to yield consistent results located in euchromatin in other Meliponini species by Cunha et al. [19].

The mapping of Fsch-C_0_*t* and (GA)_15_ probes was performed by FISH following the protocol of Pinkel et al. [28] with modifications described by Teixeira et al. [29]. Metaphase chromosomes were denatured in 70% formamide/2 × SSC at 75 °C for 5 min. After, 20 µl of hybridization mix (200 ng of labelled probe, 2 × SSC, 50% formamide, and 10% dextran sulfate) was denatured for 10 min at 85 °C and added on chromosome preparations. The slides were then incubated in a humidified chamber at 37 °C overnight (approximately 17 h) to allow for probe hybridization. Post-hybridization, the coverslips were carefully removed, and the slides were washed in 2 × SSC for 5 min, followed by a second wash in 1 × SSC for 5 min, both at room temperature with constant shaking. Slides with Fsch-C_0_*t* probe were incubated in a detection solution containing anti-digoxygenin-rhodamine for 1 h in a humid chamber at 37 °C. Then, the slides were washed in 4 × SSC/Tween at room temperature for 5 min, followed by two washes in 1 × PBS. Subsequently, the slides were dehydrated in an alcohol series 50%, 70%, and 100%. Lastly, the chromosomes were counterstained with DAPI (DAPI Fluoroshield, Sigma Aldrich). In the case of the probes (GA)_15_, the hybridization mix, incubation conditions, and post-hybridization washes followed the same protocol described above. However, as this probe was directly labeled, the antibody incubation step on the second day was omitted.

At least 30 metaphases from three individuals representing one colony per species and per probe were analyzed to determine the karyotypic patterns obtained with the two repetitive probes. The metaphases were photographed using an epifluorescence microscope Olympus BX53F attached to an Olympus XM10 camera, and CellSens image capture software. The filters WU (330–385 nm) and WG (510–550 nm) were used to analyze the fluorochromes DAPI and rhodamine and Cy3, respectively. Images were analyzed and processed using Adobe Photoshop^®^ CS6 software. Because the camera captured monochromatic images, color was added in images, and only contrast and brightness adjustments were applied.

AT-rich heterochromatin (DAPI^+^) has also been reported in several Meliponini genera [4, 30, 31, 32]. Thus, DAPI fluorochrome staining was used in *L. limao* as it may suggest the heterochromatin distribution patterns on its chromosomes.

## Results

**Fig. 1:**
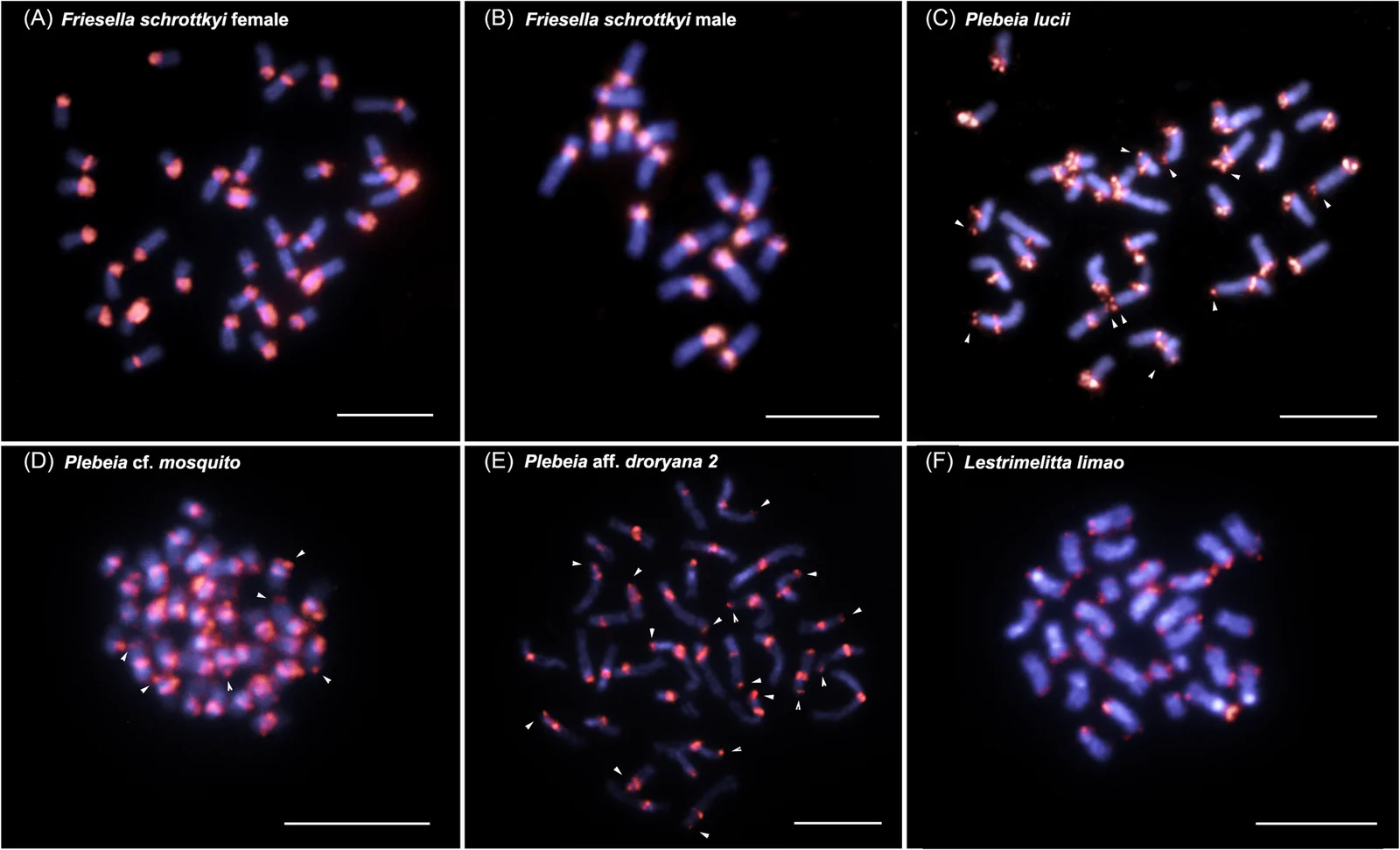
Fluorescence *in situ* hybridization using FSch-C_0_*t* probe (moderately and highly repetitive DNA) isolated from *Friesella schrottkyi* genome (red regions) and counterstained with DAPI (blue regions) in different stingless bee species: (**A**) and (**B**) *F*. *schrottkyi* (2n = 34, and *n* = 17, respectively), (**C**) *Plebeia lucii* (2n = 34), (**D**) *Plebeia* cf. *mosquito* (2n = 34), (**E**) *Plebeia* aff. *droryana* 2 (2n = 34), and (**F**) *Lestrimelitta limao* (2n = 28). Arrowheads indicate terminal markings with the FSch-C_0_*t* probe on the chromosomes of different *Plebeia* species. Bars = 5 μm

**Fig. 2:**
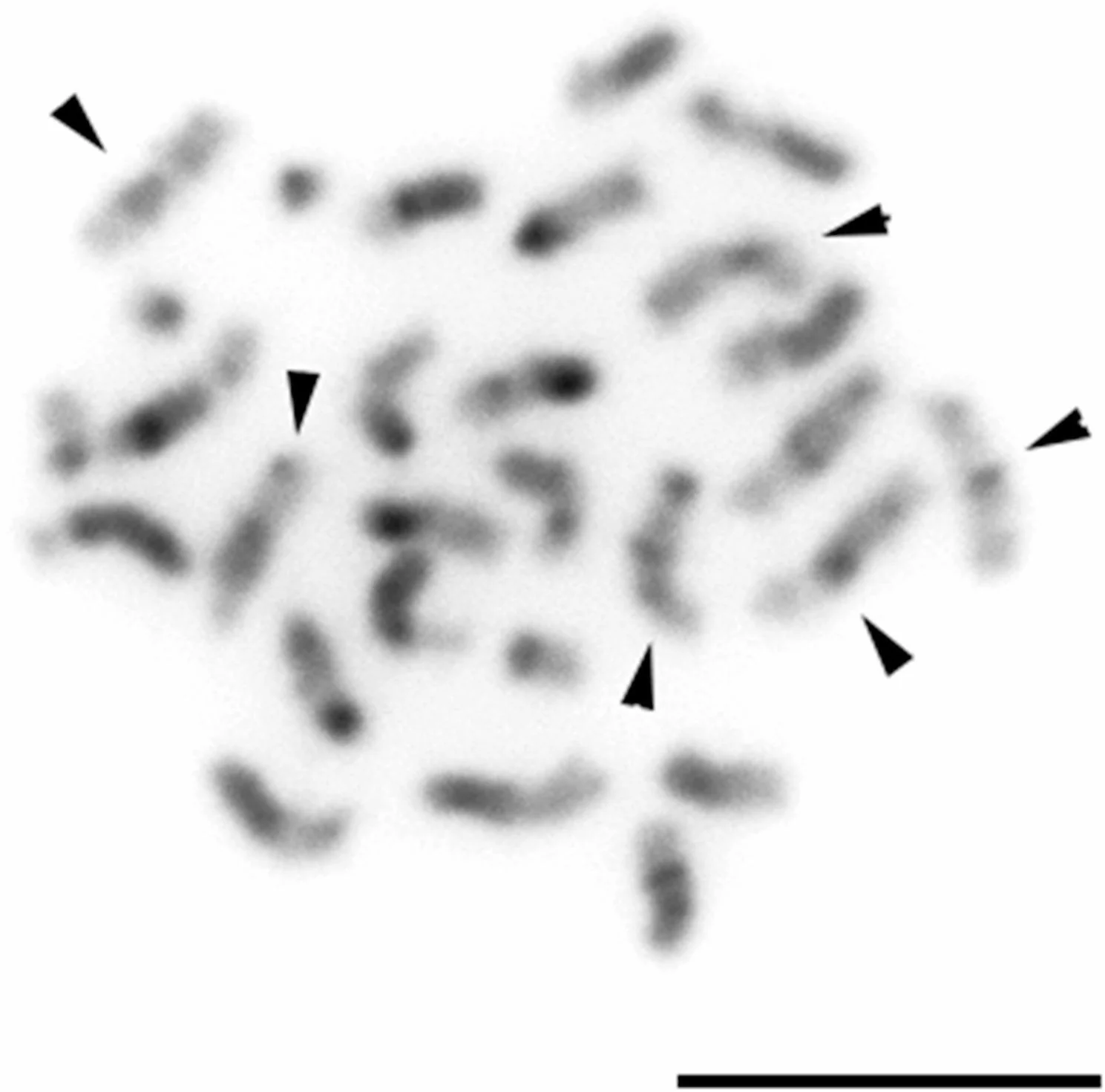
AT-rich region distribution pattern (dark regions) in mitotic metaphase of *Lestrimelitta limao* (2n = 28) using the DAPI fluorochrome staining. AT-rich regions likely correspond to heterochromatin. Arrowheads indicate chromosomes with AT-rich regions restricted to the pericentromeric regions. Bar = 5 μm

The Fsch-C_0_*t* probe labelled the pericentromeric regions of all chromosomes and the entire length of one chromosomal arm in most pairs in *F. schrottkyi*, corresponding to the heterochromatic region (Figs. 1A and B). In *P. lucii*, *P*. cf. *mosquito*, and *P*. aff. *droryana* 2, the Fsch-C_0_*t* probe revealed positive signals in the centromeric regions of all chromosomes and in the terminal regions of one or both arms of five, three, and eight pairs, respectively (Figs. 1C, D and E). In *L. limao*, this probe was detected only in the terminal regions of most chromosomes (Fig. 1F). AT-rich regions (DAPI^+^) in *L. limao* was observed in one arm (either short or long) of most chromosomes, except for three metacentric pairs, in which it was restricted to pericentromeric regions (Fig. 2). This pattern likely corresponds to the heterochromatin distribution in this species.

**Fig. 3:**
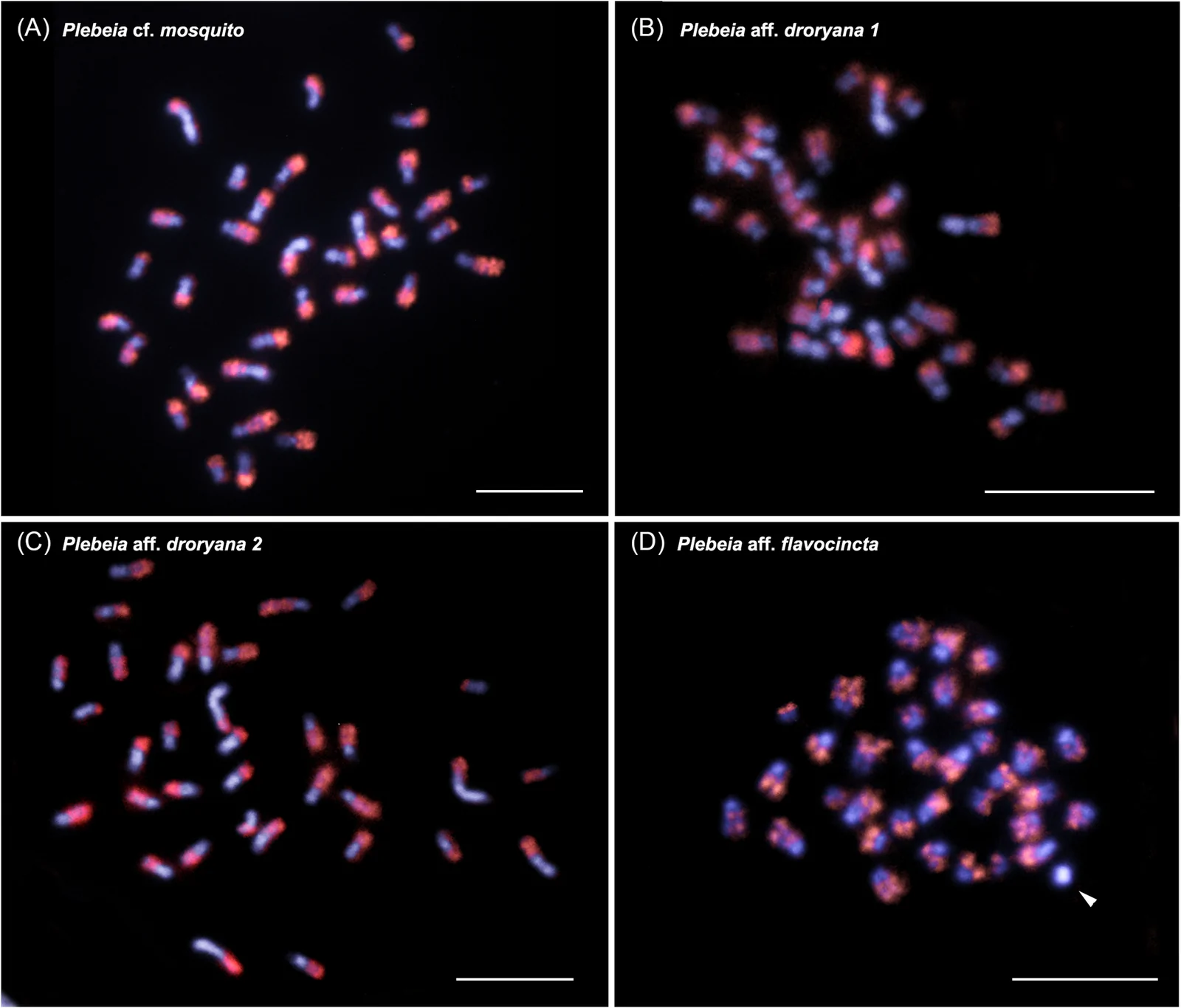
Fluorescence *in situ* hybridization with microsatellite (GA)_*15*_ probe (red regions) and counterstained with DAPI (blue regions) in different *Plebeia* species: (**A**) *Plebeia* cf. *mosquito* (2n = 34), (**B**) *Plebeia* aff. *droryana* 1 (2n = 34), (**C**) *Plebeia* aff. *droryana* 2 (2n = 34), and (**D**) *Plebeia* aff. *flavocincta* (2n = 34 + 1B). Arrowhead indicates B chromosome. Bars = 5 μm

The microsatellite (GA)_*n*_ was located on one of the chromosome arms in *Plebeia* cf. *mosquito* (Fig. 3A), *Plebeia* aff. *droryana* 1 (Fig. 3B), *Plebeia* aff. *droryana* 2 (Fig. 3C), and *Plebeia* aff. *flavocincta* (Fig. 3D), corresponding to the euchromatic region.

**Fig. 4:**
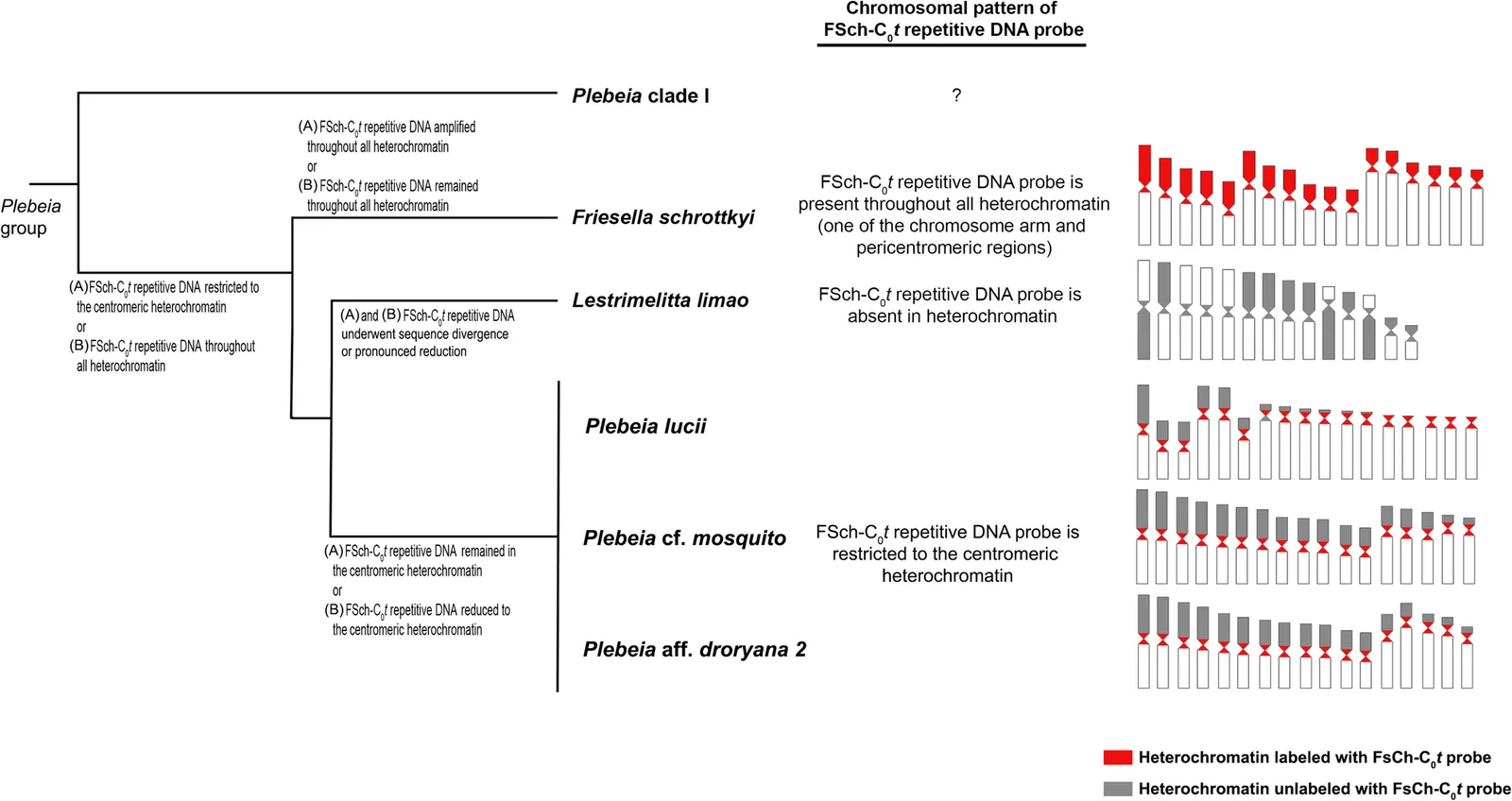
Data on the chromosomal location of the FSch-C_0_*t* probe in the heterochromatin of some *Plebeia* species from clade II (*P. lucii*, *P*. cf. *mosquito*, and *P.* aff. *droryana* 2), *Lestrimelitta limao*, and *Friesella schrottkyi* associated with their phylogenetic relationships according to Werneck [13] and Lepeco et al. [14]. We suggest two scenarios (**A** or **B**) regarding the evolution of heterochromatin composition in the group comprising *Friesella*, *Lestrimelitta* and *Plebeia* clade II. The question mark indicates that there is no data on the chromosomal localization pattern of the FSch-C_0_*t* probe in *Plebeia* clade I

Our cytogenetic data on the Fsch-C_0_*t* probe were plotted onto the phylogenetic tree drawn based on the molecular analysis of Werneck [13] and Lepeco et al. [14] (Fig. 4). We suggested two scenarios (A or B) regarding the evolution of the heterochromatin composition in the group comprising *Friesella*, *Lestrimelitta* and *Plebeia* clade II in light of available phylogenies. In the first (A), these sequences were originally restricted to centromeric heterochromatin as observed in some *Plebeia* species from clade II and were amplified in *F. schrottkyi* and may have undergone divergence and/or quantitative reduction in *L*. *limao*. In the second (B), the ancestral karyotype contained these sequences throughout the heterochromatin, with subsequent reduction to centromeric regions in *Plebeia* species from clade II and potential sequence divergence and/or more pronounced quantitative reduction in *L. limao*. No hybridization data were obtained for *Plebeia* species from clade I due to the unavailability of cytogenetic material.

## Discussion

The chromosomal distribution pattern of the Fsch-C_0_*t* probe observed in *F. schrottkyi* corresponded to the heterochromatic regions, previously analyzed by Elizeu et al. [18]. This result is consistent with the known composition of constitutive heterochromatin, which is primarily composed of repetitive sequences, including satDNA and transposable elements (TE) [3, 33, 34]. A similar pattern of heterochromatic composition, consisting of moderately and highly repetitive sequences, has also been reported in other Meliponini species [5, 6, 7, 8, 9, 10, 11] as well as other insects [20, 22, 35, 36, 37]. However, repetitive sequences are not always restricted to heterochromatic regions, as observed in some insects in which satDNAs are widely dispersed throughout the euchromatin [38, 39, 40, 41].

Overall, the data reveal the first evidence of shared repetitive sequences between *F. schrottkyi* and some *Plebeia* species from clade II within parts of the heterochromatin, contrasting with the heterochromatin divergence found in *L. limao*, as discussed below. The FISH data using the Fsch-C_0_*t* probe, combined with the C-banding [17, 42], indicated that only the centromeric heterochromatin of analyzed *Plebeia* species from clade II shared repetitive sequences with the heterochromatin of *F. schrottkyi*. However, the remaining heterochromatic regions on the short arms of *Plebeia* chromosomes had a DNA composition distinct from that of *F. schrottkyi*, indicating diversification events in portions of heterochromatin between the two genera. Since we did not include samples of *Plebeia* species from clade I, it is not possible to determine whether these patterns reflect the polyphyly of *Plebeia* or to evaluate potential cytotaxonomic markers between clades I and II. Nonetheless, divergent sequences in the short arm heterochromatin of *Plebeia* clade II may serve as cytotaxonomic markers and mirror the subclades identified within this group [13]. Future studies could focus on characterizing these sequences using genomic approaches.

Furthermore, an absence of positive signals with Fsch-C_0_*t* probe was observed in the AT-rich regions (DAPI^+^) in *L. limao*, which likely correspond to heterochromatic regions of this species. This inference is supported by the fact that microsatellites, which are generally located in euchromatic regions in Meliponini [19], exhibit a complementary distribution pattern relative to the AT-rich regions observed in *L. limao*. Thus, our data indicate a variation in the heterochromatic composition in *L. limao* compared to the karyotypes of *F. schrottkyi* and analyzed *Plebeia* species from clade II, despite their close relationship. *Lestrimelita* species exhibit peculiar biological traits compared to other Meliponini, including related genera *Friesella* and *Plebeia*, such as a diploid chromosome number of 2n = 28 [15] and obligate cleptobionts behaviour, where they sustain their nests by “plundering” resources from other Meliponini species [43, 44]. Together, these cytogenetic and behavioral differences reinforce the hypothesis of a divergent evolutionary history in *Lestrimelitta*.

By combining the FISH data with the FSch-C_0_*t* probe and available molecular phylogenies [13, 14] (see Fig. 4), we proposed two alternative scenarios regarding the evolution of heterochromatin composition in the clade comprising *Friesella*, *Lestrimelitta* and *Plebeia* clade II. *Plebeia* cf. *mosquito* and *Plebeia* aff. *droryana* 2 were not included in the molecular phylogenies, but we hypothesize they belong to *Plebeia* clade II, as the type specimen of the genus, *Plebeia mosquito*, and *Plebeia droryana* are considered part of clade II, while *Plebeia* clade I is more restricted to the Amazon biome, Central America, and North America [13, 14]. In the first hypothesis (A), FSch-C_0_*t* repetitive sequences were present in the centromeric heterochromatin of the ancestral karyotype of the clade, and this pattern was retained in some *Plebeia* species from clade II. However, some of these sequences were amplified throughout the entire heterochromatin in *F. schrottkyi.* In contrast, in *L. limao*, they may have undergone divergence and/or quantitative reduction, thereby decreasing the specificity of the FSch-C_0_*t* probe and preventing effective hybridization or detection by FISH. Alternatively, in the second hypothesis (B), FSch-C_0_*t* repetitive sequences were present throughout the entire heterochromatin in the ancestral karyotype of the clade, and this pattern was retained in *F. schrottky*. However, these sequences underwent a quantitative reduction in some *Plebeia* species from clade II, becoming restricted to centromeric heterochromatin whereas in *L. limao* they may have experienced divergence and/or a more pronounced quantitative reduction. Given the lack of data for *Plebeia* species from clade I and genera closely related to the *Plebeia* group, such as *Nannotrigona*, *Schwarziana* or *Mourella*, any inference about which of these hypotheses is more plausible would remain highly speculative.

Moreover, the repetitive DNA sequences isolated using the C_0_*t*-DNA technique from the genome of *F. schrottkyi* in this study could be older and may exist in the heterochromatin of the ancestral karyotype of the *Plebeia* group and other Meliponini genera. Supporting this observation, the satDNA family FvarSat01-301 is shared in different Meliponini genera and was found in heterochromatic regions of *F. schrottkyi* [11]. Therefore, this satDNA could be part of the repetitive sequence pool contained in the Fsch-C_0_*t* probe. However, neither *Plebeia* nor *Lestrimelitta* were analyzed by Vignati et al. [11], and the presence of this satDNA in these genera remains to be determined.

Fsch-C_0_*t* terminal signals were observed in euchromatic and/or heterochromatic chromosome arms of some chromosomes of *Plebeia* species, and *L. limao*. These sequences may potentially correspond to telomeric DNA. Repetitive telomeric DNA can be recovered using the renaturation kinetics technique and may be present in a pool of repetitive sequences within the C_0_*t*-DNA probe [45, 46, 47, 48]. The telomeric repetitive motif (TTAGG)_*n*_ has been identified in the telomeres of stingless bees [19] and is considered ancestral in insects [49, 50]. Within Hymenoptera, the (TTAGG)_n_ motif also constitutes the telomeres of ants [29]. However, wasps exhibit remarkable diversity, with telomeric repeats of 1, 6, 8, 10, 11, and 17 bp. In addition, some species lack a canonical motif, suggesting a telomerase-independent mechanism [51, 52].

Microsatellite (GA)_*n*_ was located only in one chromosomal arm of the four *Plebeia* taxa analyzed. As expected, this pattern is consistent with the chromosomal distribution of microsatellites observed in other *Plebeia* spp. and stingless bee species with *n* = 17 chromosomes within the same phylogenetic clade (encompassing most Neotropical species), likely representing an ancestral pattern. In contrast, species with derived karyotypes and low chromosome number (*n* = 8, 9, and 15) have this repetitive sequence on both arms of some chromosomes, likely reflecting fusion events [19]. Additionally, this microsatellite was located only on the euchromatin of the chromosomes of the four *Plebeia* taxa studied here, a pattern also observed in stingless bees, including *F. schrottkyi* [18], *L. limao* [19], and other insects such as ants [29, 53], wasps [54, 55], grasshoppers [56, 57] and crickets [58]. In contrast, in stink bug species such as *Triatoma infestans*, the (GA)_*n*_ repeat hybridized to heterochromatic regions on autosomal pairs and the Y chromosome, whereas in *Rhodnius prolixus* and *R. ecuadoriensis*, this microsatellite was absent from the chromosomes [59]. These observations highlight the evolutionary plasticity of microsatellite distribution across insects.

In summary, the distribution of the microsatellite (GA)_*n*_ was homogeneous among the *Plebeia* species in this study, consistent with patterns observed in Meliponini. Additionally, the results revealed that repetitive sequences are conserved in parts of the heterochromatin between *F. schrottkyi* and analyzed *Plebeia* species from clade II, but may have undergone divergence and/or a more pronounced quantitative reduction in *L. limao*. Despite the lack of sampling of *Plebeia* from clade I, our data showed how cytogenetic repetitive probes can be useful to understand karyotype diversification of closely related species. Future research should focus on the cytogenetic characterization of species from *Plebeia* clade I, which are more restricted to the Amazon region, Central America, and North America [13, 14], as well as the sequences that compose the main heterochromatin of *Lestrimelitta* and *Plebeia* from clade II using genome sequencing approach. Such investigations will contribute to a deeper understanding of the evolutionary dynamics of heterochromatin in these social insects, which play pivotal roles in karyotypic evolution and species diversification.

## Supplementary Information

Below is the link to the electronic supplementary material.

Supplementary Material 1

## Data Availability

All data generated or analyzed during this study are included in this article and supplementary information. Further inquiries can be directed to the corresponding author.

## References

[1] Thakur J, Packiaraj J, Henikoff S. Sequence, chromatin and evolution of satellite DNA. Int J Mol Sci. 2021;22(9):4309. 10.3390/ijms22094309. 33919233 PMC8122249

[2] Šatović-Vukšić E, Plohl M. Satellite DNAs—From Localized to Highly Dispersed Genome Components. Genes. 2023;14:742. 10.3390/genes14030742. 36981013 PMC10048060

[3] Cabral-de-Mello DC, Palacios-Gimenez OM. Repetitive DNAs: The invisible regulators of insect adaptation and speciation. Curr Opin Insect Sci. 2025;67:101295. . 10.1016/j.cois.2024.101295 39521343

[4] Lopes DM, Fernandes A, Diniz D, Scudeler PES, Foresti F, Campos LAO. Similarity of heterochromatic regions in the stingless bees (Hymenoptera: Meliponini) revealed by chromosome painting. Caryologia. 2014;67(3):222–6. 10.1080/0144235X.2014.974349.

[5] Piccoli MCA, Bardella VB, Cabral-de-Mello DC. Repetitive DNAs in * Melipona scutellaris* (Hymenoptera: Apidae: Meliponini): chromosomal distribution and test of multiple heterochromatin amplification in the genus. Apidologie. 2018;49(4):497–504. . 10.1007/s13592-018-0577-z

[6] Cunha MS, Campos LAO, Lopes DM. Insights into the heterochromatin evolution in the genus *Melipona* (Apidae: Meliponini). Insectes Soc. 2020;67(3):391–8. 10.1007/s00040-020-00773-6.

[7] Pereira JA, Salomão TMF, Lopes DM. Different repetitive DNA sequences make up heterochromatin in Meliponini. Apidologie. 2020;51(5):855–60. 10.1007/s13592-020-00766-1.

[8] Pereira JA, Travenzoli NM, Oliveira MP, Werneck HA, Salomão TMF, Lopes DM. Molecular cytogenetics in the study of repetitive sequences helping to understand the evolution of heterochromatin in *Melipona* (Hymenoptera, Meliponini). Genetica. 2021;149(1):55–62. 10.1007/s10709-020-00111-5. 33449238

[9] Pereira JA, Milani D, Ferretti ABS, Bardella VB, Cabral-de-Mello DC, Lopes DM. The extensive amplification of heterochromatin in *Melipona* bees revealed by high throughput genomic and chromosomal analysis. Chromosoma. 2021;130(4):251–62. 10.1007/s00412-021-00764-x. 34837120

[10] Pereira JA, Cabral-de-Mello DC, Lopes DM. The Satellite DNAs Populating the Genome of *Trigona hyalinata* and the Sharing of a Highly Abundant satDNA in *Trigona* Genus. Genes. 2023;14(2):418. 10.3390/genes14020418. 36833345 PMC9957317

[11] Vignati Z, Teixeira GA, Cunha MS, Pereira JA, Lopes DM. Cytogenomics of *Frieseomelitta varia* (Hymenoptera: Apidae) and the sharing of a satellite DNA family in several Neotropical Meliponini genera. Genes. 2025;16(1):86. . 10.3390/genes16010086 39858633 PMC11764717

[12] Rasmussen C, Cameron SA. Global stingless bee phylogeny supports ancient divergence, vicariance, and long distance dispersal. Biol J Linn Soc. 2010;9(1):206–32. 10.1111/j.1095-8312.2009.01341.x.

[13] Werneck HA. Sistemática molecular e evolução do clado *Plebeia*: análises baseadas em filogenia e datação molecular da tribo Meliponini. 2016;Thesis, Brazil.

[14] Lepeco A, Branstetter MG, Melo GA, Freitas FV, Tobin KB, Gan J, et al. Phylogenomic insights into the worldwide evolutionary relationships of the stingless bees (Apidae, Meliponini). Mol Phylogenet Evol. 2024;201:108219. 10.1016/j.ympev.2024.108219. 39414098

[15] Cunha MS, Cardoso DC, Cristiano MP, Campos LAC, Lopes DM. The bee chromosome database (Hymenoptera: Apidae). Apidologie. 2021;52(2):493–502. 10.1007/s13592-020-00838-2.

[16] Andrade BL, Lopes ALG, Teixeira GA, Tavares MG. Karyotypes and chromosomal mapping of some repetitive DNAs in two stingless bee species (Apidae: Meliponini), with the description of a B chromosome in *Plebeia* genus. Cytogenet Genome Res. 2024;164(5–6):267–75. 10.1159/000542295. 39467527

[17] Campos CL, Teixeira GA, Lopes DM, Bitencourt JA, Bezerra DD, Alves RMO, Werneck HA, Waldschmidt AM. New patterns of polymorphism in the karyotypic analysis of the genus *Plebeia* (Hymenoptera, Apidae). Apidologie. 2024;55(4):1–12. 10.1007/s13592-024-01090-8.

[18] Elizeu AM, Travenzoli NM, Ferreira RP, Lopes DM, Tavares MG. Comparative study on the physical mapping of ribosomal genes and repetitive sequences in *Friesella schrottkyi* (Friese 1900) (Hymenoptera: Apidae, Meliponini). Zool Anz. 2021;292:225–30. 10.1016/j.jcz.2021.04.006.

[19] Cunha MS, Garcia MVB, Campos LAO, Lopes DM. Cytotaxonomy and karyotype evolution in Neotropical Meliponini (Hymenoptera: Apidae) inferred by chromosomal mapping of 18S rDNA and five microsatellites. J Apic Res. 2024;63(1):208–218. 10.1080/00218839.2023.2179228

[20] Mello LRA, Tasior D, Goll LG, Artoni RF, Vicari MR, Nogaroto V, Almeida MC. Physical map of repetitive DNA and karyotype evolution in three species of the genus *Omophoita* (Coleoptera: Alticinae). Ital J Zool. 2014;81(1):16–24. 10.1080/11250003.2014.882995.

[21] Terencio ML, Schneider CH, Gross MC, Carmo EJ, Nogaroto V, Almeida MC, et al. Repetitive sequences: the hidden diversity of heterochromatin in prochilodontid fish. Comp Cytogen. 2015;9(4):465–81. 10.3897/CompCytogen.v9i4.5299. PMC4698564 26752156

[22] Anjos A, Rocha GC, Paladini A, Mariguela TC, Cabral-de-Mello DC. Karyotypes and repetitive DNA evolution in six species of the genus *Mahanarva* (Auchenorrhyncha: Cercopidae). Cytogenet Genome Res. 2016;149(4):321–327. 10.1159/000450730 27811473

[23] Carvalho ND, Carmo E, Neves RO, Schneider CH, Gross MC. Differential repetitive DNA composition in the centromeric region of chromosomes of Amazonian lizard species in the family Teiidae. Comp Cytogenet. 2016;10(2):203–17. 10.3897/CompCytogen.v10i2.7081. 27551343 PMC4977797

[24] Lima JF, Carvalho LS, Carvalho MA, Schneider MC. Chromosome diversity in Buthidae and Chactidae scorpions from Brazilian fauna: Diploid number and distribution of repetitive DNA sequences. Genet Mol Biol. 2023;46(2):e20220083. 10.1590/1678-4685-GMB-2022-0083. 37216321 PMC10202225

[25] Imai H, Taylor RW, Crosland MW, Crozier RH. Modes of spontaneous chromosomal mutation and karyotype evolution in ants with reference to the minimum interaction hypothesis. Jpn J Genet. 1988;63:159–85. 10.1266/jjg.63.159. 3273765

[26] Zwick MS, Hanson RE, Islam-Faridi MN, Stelly DM, Wing RA, Price HJ, et al. A rapid procedure for the isolation of C0t-1 DNA from plants. Genome. 1997;40(1):138–42. 10.1139/g97-020. 18464813

[27] Waldschmidt AM, Salomão TMF, Barros EGD, Campos LAO. Extraction of genomic DNA from *Melipona quadrifasciata* (Hymenoptera: Apidae, Meliponinae). Braz J Genet. 1997;20:421–3. 10.1590/S0100-84551997000300011.

[28] Pinkel D, Straume T, Gray JW. Cytogenetic analysis using quantitative, high-sensitivity, fluorescence hybridization. PNAS USA. 1986;83:2934–8. 10.1073/pnas.83.9.2934. 3458254 PMC323421

[29] Teixeira GA, Barros LAC, Aguiar HJAC, Lopes DM. Multiple heterochromatin diversification events in the genome of fungus-farming ants: insights from repetitive sequences. Chromosoma. 2022;131(1):59–75. 10.1007/s00412-022-00770-7. 35325297

[30] Domingues AMT, Waldschmidt AM, Andrade SE, Andrade-Souza V, Alves RMDO, Silva Junior JCD, Costa MA. Karyotype characterization of *Trigona fulviventris* Guérin, 1835 (Hymenoptera, Meliponini) by C banding and fluorochrome staining: Report of a new chromosome number in the genus. Genet Mol Biol. 2005;28(3):390–3. 10.1590/S1415-47572005000300009.

[31] Godoy DC, Ferreira RP, Lopes DM. Chromosomal variation and cytogenetics of *Plebeia lucii *and *P. phrynostoma* (Hymenoptera: Apidae). Fla Entomol. 2013;96(4):1559–66. 10.1653/024.096.0439

[32] Cunha MS, Travenzoli NM, Ferreira RP, Cassinela EK, Silva H, Salomão TMF, Lopes DM. Comparative cytogenetics in three *Melipona* species (Hymenoptera: Apidae) with two divergent heterochromatic patterns. Genet Mol Biol. 2018;41(4):806–13. 10.1590/1678-4685-GMB-2017-0330 30508005 PMC6415597

[33] Grewal SI, Jia S. Heterochromatin revisited. Nat Rev Genet. 2007;8(1):35–46. 10.1038/nrg2008. 17173056

[34] Nishibuchi G, Déjardin J. The molecular basis of the organization of repetitive DNA-containing constitutive heterochromatin in mammals. Chromosome Res. 2017;25(1):77–87. 10.1007/s10577-016-9547-3. 28078514

[35] Cabral-de-Mello DC, Moura RDC, Melo AS, Martins C. Evolutionary dynamics of heterochromatin in the genome of *Dichotomius* beetles based on chromosomal analysis. Genetica. 2011;139(3):315–25. 10.1007/s10709-011-9551-7. 21267635

[36] Bardella VB, da Rosa JA, Vanzela ALL. Origin and distribution of AT-rich repetitive DNA families in *Triatoma infestans* (Heteroptera). Infect Genet Evol. 2014;23:106–14. 10.1016/j.meegid.2014.01.035. 24524986

[37] Palacios-Gimenez OM, Carvalho CR, Soares FAF, Cabral-de-Mello DC. Contrasting the chromosomal organization of repetitive DNAs in two Gryllidae crickets with highly divergent karyotypes. PLoS ONE. 2015;10(12):e0143540. 10.1371/journal.pone.0143540. 26630487 PMC4667936

[38] Montiel EE, Panzera F, Palomeque T, Lorite P, Pita S. Satellitome analysis of *Rhodnius prolixus*, one of the main Chagas disease vector species. Int J Mol Sci. 2021;22(11):6052. 10.3390/ijms22116052. 34205189 PMC8199985

[39] Montiel EE, Mora P, Rico-Porras JM, Palomeque T, Lorite P. Satellitome of the red palm weevil, *Rhynchophorus ferrugineus* (Coleoptera: Curculionidae), the most diverse among insects. Front Ecol Evol. 2022;10:826808. 10.3389/fevo.2022.826808.

[40] Cabral-de‐Mello DC, Mora P, Rico‐Porras JM, Ferretti AB, Palomeque T, Lorite P. The spread of satellite DNAs in euchromatin and insights into the multiple sex chromosome evolution in Hemiptera revealed by repeatome analysis of the bug *Oxycarenus hyalinipennis*. Insect Mol Biol. 2023;32(6):725–37. 10.1111/imb.12868. 37615351

[41] Rico-Porras JM, Mora P, Palomeque T, Montiel EE, Cabral-de-Mello DC. Lorite P. Heterochromatin is not the only place for satDNAs: the high diversity of satDNAs in the euchromatin of the beetle *Chrysolina americana* (Coleoptera, Chrysomelidae). Genes. 2024;15(4):395. 10.3390/genes15040395 38674330 PMC11049206

[42] 42 Godoy DC, Ferreira RP, Lopes DM. Chromosomal variation and cytogenetics of *Plebeia lucii* and *P. phrynostoma* (Hymenoptera: Apidae). Fla Entomol. 2013;96(4):1559–66. 10.1653/024.096.0439.

[43] Marchi P, Melo GA. Revisão taxonômica das espécies brasileiras de abelhas do gênero *Lestrimelitt*a Friese (Hymenoptera, Apidae, Meliponina). Rev Bras Entomol. 2006;50:6–30. 10.1590/S0085-56262006000100002.

[44] Engel MS, Rasmussen C, Ayala R, Oliveira FF. Stingless bee classification and biology (Hymenoptera, Apidae): a review, with an updated key to genera and subgenera. ZooKeys. 2023;1172:239. 10.3897/zookeys.1172.104944. 37547181 PMC10401200

[45] Chang SB, Yang TJ, Datema E, Van Vugt J, Vosman B, Kuipers A, et al. FISH mapping and molecular organization of the major repetitive sequences of tomato. Chromosome Res. 2008;16:919–33. 10.1007/s10577-008-1249-z. 18688733

[46] Begum R, Alam SS, Menzel G, Schmidt T. Comparative molecular cytogenetics of major repetitive sequence families of three *Dendrobium* species (Orchidaceae) from Bangladesh. Ann Bot. 2009;104(5):863–72. 10.1093/aob/mcp178. 19635741 PMC2749531

[47] Zakrzewski F, Wenke T, Holtgräwe D, Weisshaar B, Schmidt T. Analysis of a c0t-1 library enables the targeted identification of minisatellite and satellite families in *Beta vulgaris*. BMC Plant Biol. 2010;10:1–14. 10.1186/1471-2229-10-8. 20064260 PMC2820488

[48] Cuyacot AR, Won SY, Park SK, Sohn SH, Lee J, Kim JS, et al. The chromosomal distribution of repetitive DNA sequences in *Chrysanthemum boreale* revealed a characterization in its genome. Sci Hortic. 2016;198:438–44. 10.1016/j.scienta.2015.12.025.

[49] Vítková M, Král J, Traut W, Zrzavý J, Marec F. The evolutionary origin of insect telomeric repeats (TTAGG)n. Chromosome Res. 2005;13(2):145–56. 10.1007/s10577-005-7721-0. 15861304

[50] Menezes RS, Bardella VB, Cabral-de-Mello DC, Lucena DA, Almeida EA. Are the TTAGG and TTAGGG telomeric repeats phylogenetically conserved in aculeate Hymenoptera? Sci Nat. 2017;104(9):85. 10.1007/s00114-017-1507-z. 28956077

[51] Fajkus P, Adámik M, Nelson AD, Kilar AM, Franek M, Bubeník M, et al. Telomerase RNA in Hymenoptera (Insecta) switched to plant/ciliate-like biogenesis. Nucleic Acids Res. 2023;51(1):420–33. 10.1093/nar/gkac1202. 36546771 PMC9841428

[52] Lukhtanov VA, Pazhenkova EA. Diversity and evolution of telomeric motifs and telomere DNA organization in insects. Biol J Linn Soc. 2023;140(4):536–55. 10.1093/biolinnean/blad068.

[53] Silveira LI, Teixeira GA, Barros LAC, Dergam JA, Aguiar HJAC. Chromosomal diversity in *Crematogaster* Lund, 1831 (Formicidae: Myrmicinae) from the Amazon rainforest. Genome. 2024;68:1–12. 10.1139/gen-2023-0130. 39226616

[54] Marchioro P, Campos LAO, Lopes DM. First record of a B chromosome in *Polybia fastidiosuscula* Saussure (Vespidae) and investigation of chromatin composition through microsatellite mapping. Cytogenet Genome Res. 2020;160(11–12):711–8. 10.1159/000513641. 33752199

[55] Tavares MG, Teixeira GA. Cytogenetic characterization of solitary wasp *Ancistrocerus flavomarginatus* (Brèthes, 1906) (Hymenoptera, Vespidae) with insights into the chromosomal evolution in the genus. Genome. 2023;66(3):62–7. 10.1139/gen-2022-0095. 36645884

[56] Milani D, Cabral-de-Mello DC. Microsatellite organization in the grasshopper *Abracris flavolineata* (Orthoptera: Acrididae) revealed by FISH mapping: remarkable spreading in the A and B chromosomes. PLoS ONE. 2014;9(5):e97956. 10.1371/journal.pone.0097956. 24871300 PMC4037182

[57] Ruiz-Ruano FJ, Cuadrado Á, Montiel EE, Camacho JPM, López-León MD. Next generation sequencing and FISH reveal uneven and nonrandom microsatellite distribution in two grasshopper genomes. Chromosoma. 2015;124(2):221–34. 10.1007/s00412-014-0492-7. 25387401

[58] Palacios-Gimenez OM, Cabral-de-Mello DC. Repetitive DNA chromosomal organization in the cricket *Cycloptiloides americanus*: a case of the unusual X 1 X 2 0 sex chromosome system in Orthoptera. Mol Genet Genom. 2015;290(2):623–31. 10.1007/s00438-014-0947-9. 25373534

[59] Panzera F, Cuadrado Á, Mora P, Palomeque T, Lorite P, Pita S. Differential spreading of microsatellites in holocentric chromosomes of chagas disease vectors: genomic and evolutionary implications. Insects. 2023;14(9):772. 10.3390/insects14090772. 37754740 PMC10531928

