# Supplementary material for: Molecular cytogenetics provides insights into heterochromatin composition and karyotypic evolution in species of the Plebeia group (Apidae: Meliponini)
Source: Mol Cytogenet. 2026 Jun 16;19:34. doi: 10.1186/s13039-026-00775-2 (PMC13579972; doi:10.1186/s13039-026-00775-2)
Supplement: Supplementary file 1 [file 13039_2026_775_MOESM1_ESM.docx]

**Table S1**. Stingless bee species used in this study for the molecular cytogenetic analysis, their respective localities, diploid chromosome numbers and references that contain descriptions of their karyotypes.

| **Stingless bee species** | **Locality** | **Coordinates** | **Chromosome Number** | **References** |
| --- | --- | --- | --- | --- |
| *Friesella schrottkyi* | Viçosa, Minas Gerais, Brazil  Bahia, Brazil | 20° 45′ 32″ S, 42° 52′ 7″ W  13° 56′ 37″ S, 39° 7′ 4″ W | 2n=34 | Elizeu et al. (2021) |
| *Lestrimelitta limao* | Urbano Santos, Maranhão Brazil | 3° 12′ 26″ S, 43° 24′ 43″ W | 2n=28 | Cunha et al. (2021) |
| *Plebeia lucii* | Viçosa, Minas Gerais, Brasil | 20° 45′ 32″ S, 42° 52′ 7″ W | 2n=34 | Godoy et al. (2013) |
| *Plebeia* cf. *mosquito* | Gandu, Bahia, Brasil | 13° 49′ 11″ S – 39º 35′ 02″ W | 2n=34 | Campos et al. (2024) |
| *Plebeia* aff. *droryana* 1 | Iraquara, Bahia, Brasil | 12° 43′ 33″ S – 41° 36′ 28″ W | 2n=34 | Campos et al. (2024) |
| *Plebeia* aff. *droryana* 2 | Gandu, Bahia, Brasil | 13° 49′ 11″ S – 39º 35′ 02″ W | 2n=34 | Campos et al. (2024) |
| *Plebeia* aff. *flavocincta* | Licínio de Almeida, Bahia, Brasil | 14° 41′ 20″ S – 42° 30′ 13″ W | 2n=34 | Campos et al. (2024) |
